# Case Report: Robot-assisted sacral fracture reduction with patient-specific finite element planning

**DOI:** 10.3389/fmed.2025.1710981

**Published:** 2025-11-14

**Authors:** Yupeng Ma, Junbo Ge, Huanyu Hong, Tao Huang, Yu Li, Zhengwen Sun, Tao Sun

**Affiliations:** 1 Orthopaedics Department, Yantai Shan Hospital, Yantai, China; 2 Yantai Key Laboratory for Repair and Reconstruction of Bone & Joint, Yantai Shan Hospital, Yantai, China

**Keywords:** sacral fracture, robot-assisted fracture reduction (RAFR), finite element analysis (FEA), minimally invasive surgery, pelvic trauma

## Abstract

**Background:**

Sacral fractures are typically caused by high-energy trauma. They often disrupt the pelvic ring and pose complex anatomical challenges, as the sacrum is surrounded by critical structures—including blood vessels, nerves, and internal organs. Traditional open reduction and internal fixation (ORIF) can restore anatomical alignment but requires extensive tissue exposure. This exposure leads to greater tissue trauma, prolonged recovery, and higher risks of infection, hemorrhage, or nerve damage. For these reasons, minimally invasive surgery (MIS) is preferred. However, MIS demands high technical precision. Robot-assisted fracture reduction (RAFR) systems enhance precision in minimally invasive procedures, while finite element analysis (FEA) optimizes preoperative planning by simulating biomechanics. However, clinical evidence for combining these techniques in complex, multi-injury cases is limited.

**Case presentation:**

A 19-year-old female was admitted to the hospital following high-energy trauma (a fall from height), diagnosed with unstable pelvic fracture (AO C1.3 type), longitudinal sacral fracture (Denis II type with vertical displacement), and multiple concurrent injuries (thoracolumbar fractures, rib fracture, pulmonary contusion, splenic and renal contusions, lumbosacral plexus injury). Preoperative management included supracondylar femoral traction and vital sign stabilization. Preoperative FEA based on the patient’s CT data simulated three internal fixation schemes, showing comparable vertical stability; S1 standard + S2 extended sacroiliac screws were selected to preserve lumbar mobility. The RAFR system was used for surgery: 3D preoperative planning, automatic path design, and intraoperative real-time tracking. Fixation was performed with the selected screws (posterior ring) and an anterior external fixator.

**Conclusion:**

This case illustrates the value of combining FEA and RAFR in treating complex sacral fractures with multiple traumas. It highlights that FEA provides a scientific basis for personalized fixation strategy selection, while RAFR achieves precise, minimally invasive reduction, offering a feasible pathway for personalized, minimally invasive management.

## 1 Introduction

Sacral fractures, often from high-energy trauma like motor vehicle accidents or falls, frequently disrupt the pelvic ring and may involve the acetabulum, sacroiliac joints, and pubic symphysis. The complex sacral and pelvic anatomy, along with surrounding critical structures (blood vessels, lumbosacral plexus, internal organs), complicates treatment (1–4). Traditional open reduction and internal fixation (ORIF) restores alignment but brings extensive exposure, tissue trauma, prolonged recovery, and higher risks of infection, hemorrhage, or nerve damage (5), leading to a preference for minimally invasive surgery (MIS) to reduce trauma and speed recovery (6).

MIS for sacral fractures requires high technical skill due to anatomical complexity and individual variations (7). Robot-assisted systems offer stable, precise control for minimally invasive complex reductions, boosting accuracy, safety, and reducing complication (7). Finite element analysis (FEA) simulates stress distribution, bone–implant interactions, and treatment mechanical outcomes, optimizing preoperative plans to improve success and reduce complications.

In sacral fracture management, FEA aids in selecting optimal strategies by simulating mechanical responses (8–12), and when integrated with robotics, enhances precision via preoperative planning and intraoperative navigation. However, clinical evidence for such combinations—especially in complex, multi-injury cases—is limited.

This case report presents robotic-assisted fracture reduction (RAFR) combined with preoperative FEA in treating an unstable sacral fracture with multiple traumas, highlighting how this approach addresses minimally invasive challenges for personalized, precise management.

## 2 Case presentation

The patient is a 19-year-old female admitted to the hospital due to high-energy trauma. Physical examination on admission: vital signs were stable, pressure pain in the pelvic region was obvious, lower limb movement was limited, and sensation was reduced (suggesting lumbosacral plexus injury). Imaging examinations (pelvic orthostatic, inlet and outlet X-rays and CT) showed instability of the pelvic ring and a longitudinal fracture of the sacrum (Denis II type) with vertical displacement. The patient had no prior history of chronic illness or surgery. A final diagnosis was made as follows: (1) Pelvic fracture (AO C1.3 type); (2) lumbosacral plexus nerve injury; (3) thoracic vertebral fractures (T5, T8, T12); (4) lumbar vertebral fractures (L1, L2, L3); (5) lumbar transverse process fractures (L1, L2, L3, L5); (6) pelvic effusion; (7) first rib fracture; (8) pulmonary contusion; (9) splenic contusion; (10) renal contusion. Preoperative management: Supracondylar femoral traction was applied due to vertical instability of the pelvis. Stabilization of the patient’s vital signs and symptomatic treatment of multiple injuries throughout the body.

**Figure 1:**
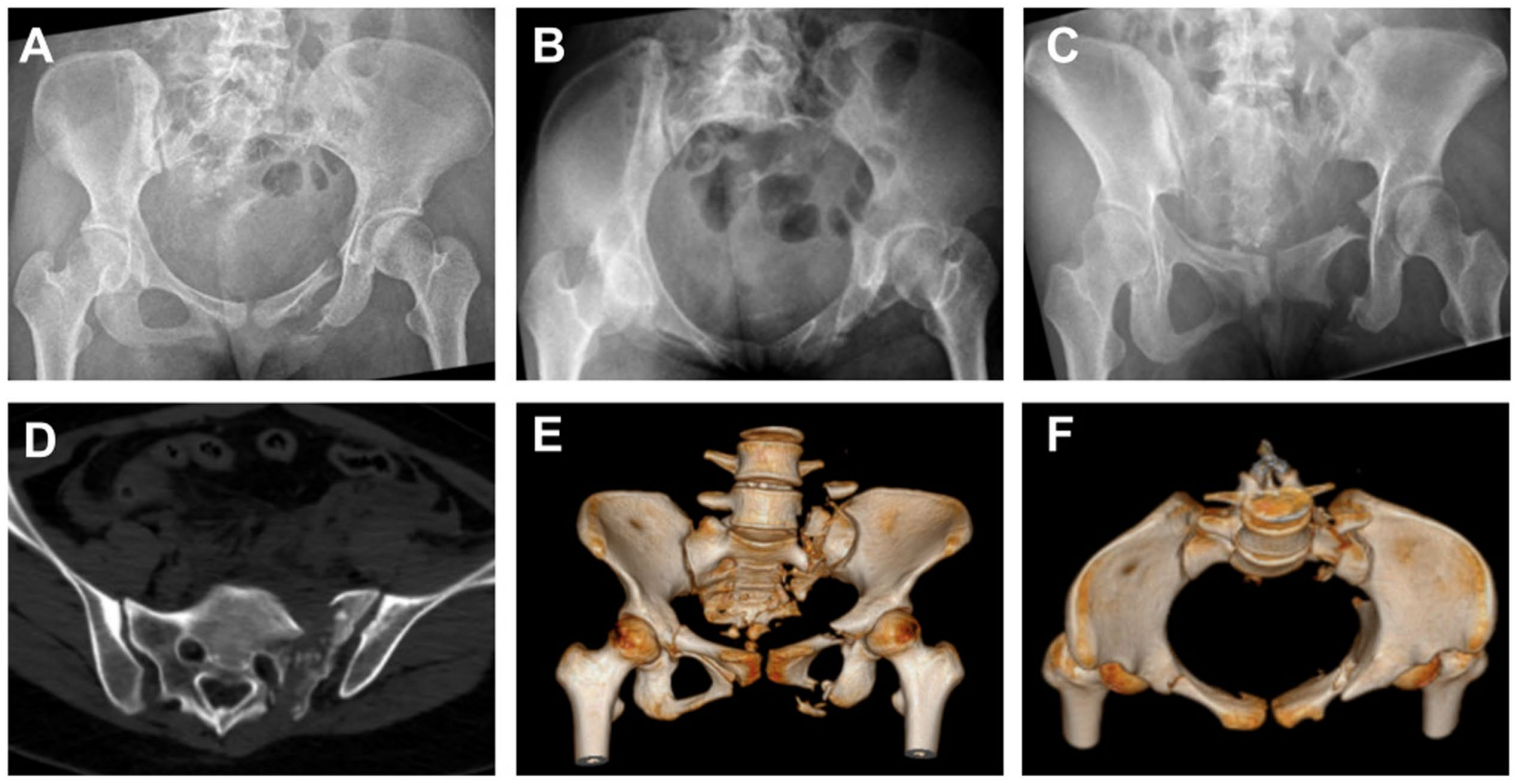
Preoperative imaging studies of the patient’s pelvis were performed using X-rays in the anteroposterior **(A)**, inlet **(B)**, and outlet views **(C)**. The findings suggested a fracture, and CT **(D)** with three-dimensional reconstruction **(E,F)** provided more detailed information.

During the preoperative assessment, anteroposterior (AP), inlet, and outlet X-rays of the pelvis were obtained, along with CT scans for a comprehensive evaluation Figure 1. For patients with vertical instability, supracondylar femoral traction was applied. The surgical plan was developed by considering the fracture type, expected reduction outcome, and the feasibility of constructing bony tunnels.

To clarify the chronological sequence of the patient’s clinical management and follow-up, key events—from admission to the 3-month follow-up—are summarized in Table 1. This timeline highlights the alignment between interventions (e.g., preoperative planning, surgery) and corresponding assessments (e.g., imaging, clinical evaluations).

**Table 1:** Timeline of key clinical events for the patient from admission to 3-month follow-up.

Time node	Key event type	Specific content and key findings
2024-2-24 (time: 17:00)	Injury (high-energy trauma)	The patient sustained a high-energy trauma from a fall from height, presenting with severe pelvic pain, limited lower limb movement, and reduced sensation. A preliminary on-site assessment suggested pelvic ring instability.
202X-2-24 (time: 17:00 + 8 h)	Admission	Admitted to the Orthopaedics Department of Yantai Shan Hospital. Vital signs were stabilized after emergency treatment. Physical examination confirmed obvious pelvic tenderness, positive signs of lumbosacral plexus injury (reduced lower limb sensation/movement).
2024-2-24 + 1d to 2024-2-24 + 11d	Preoperative management and planning	Applied supracondylar femoral traction to correct pelvic vertical instability. Completed pelvic AP/inlet/outlet X-rays and 64-slice spiral CT (1 mm slice thickness) for imaging evaluation. Established patient-specific finite element model, simulated three fixation schemes (SDS1EDS2, L5SDS1, L5EDS2), and confirmed SDS1EDS2 as the optimal plan (preserving lumbar mobility with equivalent vertical stability).
2024-2-24 + 12d (time: 09:10)	Surgery (RAFR-assisted fixation)	Under general anesthesia, the RAFR system was used for 3D preoperative planning, automatic path design, and real-time tracking. Completed minimally invasive reduction: placed S1 standard + S2 extended sacroiliac screws (posterior ring) and anterior inferior iliac spine external fixator (anterior ring). Intraoperative X-ray confirmed accurate screw placement and satisfactory fracture reduction. The operation lasted 150 min with minimal bleeding (<100 mL).
2024-2-24 + 12d + 3d	Postoperative short-term evaluation	CT reexamination showed fracture reduction met Matta’s “excellent” criteria; no implant malposition or loosening. The patient’s pelvic pain was significantly relieved; lower limb movement range was slightly improved (no obvious neurological deterioration).
2024-2-24 + 12d + 3d + 3 m (postoperative 3 months)	Follow-up	Clinical assessment: no pelvic pain, normal hip range of motion (0°–120° for flexion/extension), partial recovery of lower limb sensation (S1–S3 dermatome), and muscle strength (grade 4 for hip/knee flexion). Imaging evaluation: X-ray/CT showed good fracture healing (clear callus formation, blurred fracture line), no implant loosening or pelvic ring instability.

## 3 Materials and methods

### 3.1 Overview of the RAFR system

The RAFR system consists of five main components: fracture reduction software, an optical tracking device, a reduction robot, a holding device, and an elastic traction device. The system ensures precise control during the reduction process by real-time tracking of the patient’s pelvis and robotic operations. The holding and elastic traction devices stabilize the pelvis and counteract the restrictive forces from surrounding soft tissues.

### 3.2 Finite element model establishment

This study utilized CT data (64-slice spiral CT, 1 mm layer thickness) of the patient’s sacral bone L4-L5 vertebral body and pelvis. First, virtual three-dimensional models of the lumbar spine and pelvis were created from DICOM-format CT data using image processing software (Mimics 21.0), with components segmented based on CT grey values. Next, the pelvic 3D model (generated in Mimics) was imported into Design X 2020.0 software for smoothing, ensuring it was suitable for subsequent computations. Ten-node tetrahedral elements were used. The mesh model was then assigned material properties, set as heterogeneous and isotropic. Material properties were assigned to different skeletal regions based on grey values. Using Mimics’ built-in formulas, pelvic grey values were divided into 10 levels, and empirical formulas were assigned based on relevant literature (10, 13, 14). Using the design functions in SpaceClaim 2019 software, models such as intervertebral disks and cylindrical screws were drawn, and then the drawn models were moved to appropriate positions using the move command to simulate fracture reduction and fixation.

### 3.3 Ligament and muscle modeling and load application

The skeletal and screw mesh models were imported into ANSYS WORKBENCH 2020, and spring-damper elements were used to simulate ligaments and muscles. Material properties and ligament parameters were referenced from previous literature (8, 9, 15, 16). The material properties of the internal fixation were designed as titanium alloy. The sacroiliac joint and pubic symphysis were modeled using constraint constraints, with six degrees of freedom constraints applied to the nodes of both acetabula. A vertical downward force of 500 N was applied to the upper surface of the L4 vertebra. This simulated the weight-bearing effect during upright standing. The pelvic model uses spring elements to simulate the main ligament structures around the pelvis, ensuring joint mobility and stress transmission at the sacroiliac joint (8, 14). Displacement results show that the anterior margin of the sacrum has the greatest mobility, exhibiting a forward and downward movement trend, while both iliac bones exhibit a rotational trend, consistent with literature reports (9, 15). The sacroiliac joint cartilage surface is set as a sliding friction contact (friction coefficient 0.015) (17). The fracture surface is set as a sliding friction contact (friction coefficient 0.3) (18). The screw thread and bone contact surface, the connection between the screw tail and the disc, and the disc and cortical bone surface are all bound connections.

### 3.4 Internal fixation model design

**Figure 2:**
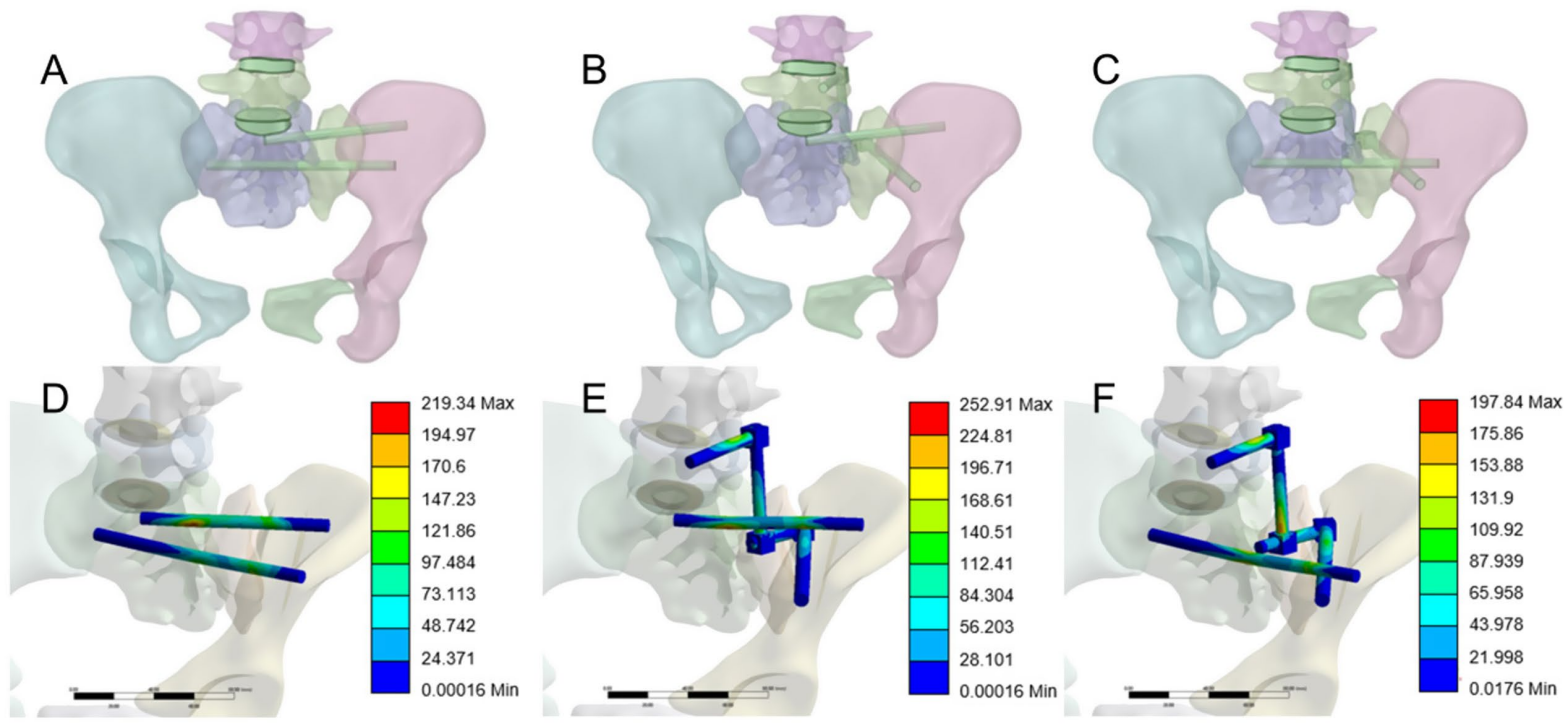
**(A)** SI standard sacroiliac screw + S2 extended sacroiliac screw (SDS 1 EDS2). **(B)** Unilateral segment iliac-lumbar fixation + SI standard sacroiliac screw (L5SDS1). **(C)** Unilateral 1.5 segment iliac-lumbar fixation + S2 extended sacroiliac screw (L5EDS2). **(D)** Maximum von Mises stress contour map of SDSIEDS2. **(E)** Maximum von Mises stress contour map of L5SDS1. **(F)** Maximum von Mises stress contour map of L5EDS2.

In this study, standard sacroiliac screws are defined as screws that traverse the fracture line and reach the midline of the sacrum. Extended sacroiliac screws are defined as screws that traverse the fracture line and pass through the contralateral ilium. Three internal fixation models were established, as shown in Figures 2A–C: (1) S1 standard sacroiliac screw + S2 extended sacroiliac screw (SDS1EDS2); (2) unilateral L5 segment iliac-lumbar fixation + S1 standard sacroiliac screw (L5SDS1); (3) unilateral L5 segment iliac-lumbar fixation + S2 extended sacroiliac screw (L5EDS2). The lengths and diameters of the lumbar pedicle screws and iliac screws were 45 mm and 6.5 mm, and 70 mm and 7.5 mm, respectively, while the diameter of the sacroiliac screw was 7.3 mm. All materials are made of titanium alloy. A Boolean operation was performed on the three fixation models, and the vertical displacement of each model was recorded.

## 4 Results

### 4.1 Implementation

#### 4.1.1 Preoperative preparation and system configuration

**Figure 3:**
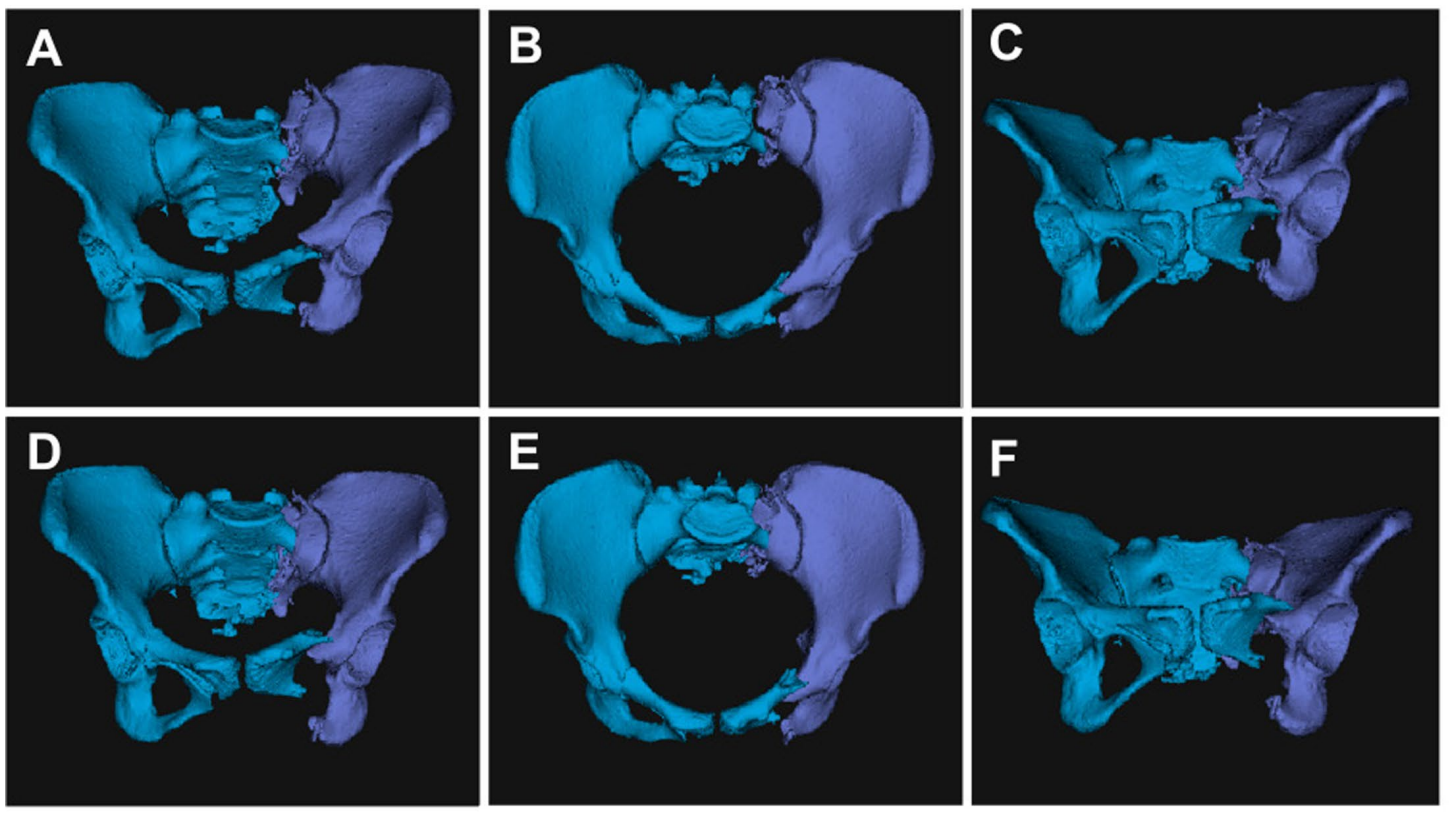
The reduction path was fine-tuned based on preoperative planning. Automatic segmentation and 3D reconstruction of pelvic images using a pelvic realignment **(A,B,C)**. Subsequently, computer-aided surgical planning was performed for the pelvic fracture **(D,E,F)**.

Preoperative CT data were integrated into the robotic system, and the pelvic fracture images were segmented using planning software to construct a reduction model based on mirror symmetry principles (19). The system utilizes an automatic reduction algorithm to perform optimal path planning, which is reviewed and adjusted by the physician to ensure safe and efficient execution (5, 20) (Figure 3).

#### 4.1.2 Surgical procedure

- The patient was positioned supine under general anesthesia, with the hip elevated for better access.
- Following the intraoperative layout, the holding arms were connected, and standard disinfection procedures were carried out.
- The patient and robotic trackers were securely installed, CBCT data were collected, and image registration was performed.
- Using navigation drilling, Schantz pins were placed under guidance to ensure pelvic stability.
- Femoral traction was applied, and the robotic arm moved the pelvis along the planned path to the target position.
- After achieving reduction, appropriate implants were selected to stabilize the pelvic structure.
- X-rays were taken before the conclusion of the procedure to verify accurate reduction and correct screw placement.

**Figure 4:**
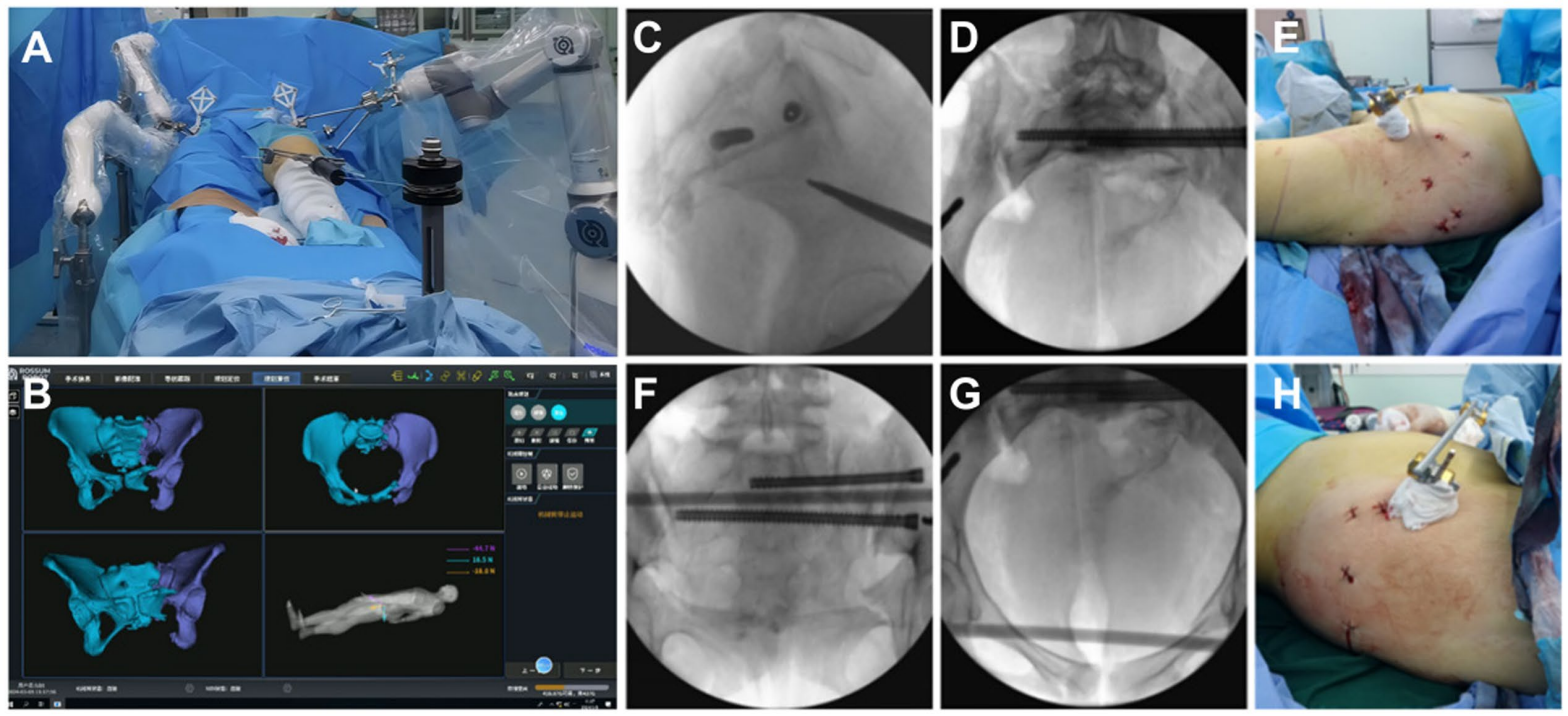
**(A)** A connection was established between the five Schantz pins and the holding device, and the femoral condylar traction pin was linked to the elastic traction device. **(B)** Under real-time 3D navigation, the robotic arm autonomously moved the affected hemipelvis along the pre-planned reduction path, achieving autonomous reduction. **(C–H)** AII screw channels were verified by fluoroscopy, and the results were satisfactory. The wound appearance was minimally invasive.

The surgical procedure is shown in Figure 4.

### 4.2 Finite element biomechanical

To assess the stability of the posterior pelvic ring fixation, we measured the displacement at five points (A-E) on the superior sacral surface under a 500 N vertical load applied to the superior endplate of the L4 vertebra. Vertical displacement data for different sacral fixation models, in Table 2. To identify significant differences in vertical displacement between the three models, we used SPSS 26.0 (IBM Corp., United States) to analyze sacral surface displacement data. First, we conducted normality tests on the sacral displacement data, including the Kolmogorov–Smirnov (V) test and Shapiro–Wilk test. These tests yielded *p*-values > 0.05 for all groups, indicating the data followed a normal distribution. Homogeneity of variance tests were conducted on the vertical displacement data of the three groups, and the results showed that the *p*-values were greater than 0.05. To further compare sacral displacement among the three groups, an analysis of variance (ANOVA) was performed, and the results showed no significant differences between groups (*p* > 0.05). Based on the statistical analysis, it was concluded that there were no significant differences in sacral vertical displacement among the SDS1EDS2, L5SDS1, and L5EDS2 groups. The three internal fixation methods were equally effective in terms of vertical stability. The maximum von Mises stress of the internal implants in the three groups was 219.34 MPa, 252.91 MPa, and 197.84 MPa, respectively, as shown in Figures 2D–F. From a safety perspective, the maximum stress values of these three internal fixation groups are far below the yield stress of titanium metal (1,050 MPa) (21).

**Table 2:** Displacement data for the superior surface of the sacrum.

Groups	A (mm)	B (mm)	C (mm)	D (mm)	E (mm)
SDS1EDS2	0.64003	0.56687	0.68932	0.73965	0.65342
L5SDS1	0.58271	0.56515	0.67418	0.69285	0.61795
L5EDS2	0.61334	0.61633	0.72263	0.73052	0.65944

### 4.3 Follow-up results

**Figure 5:**
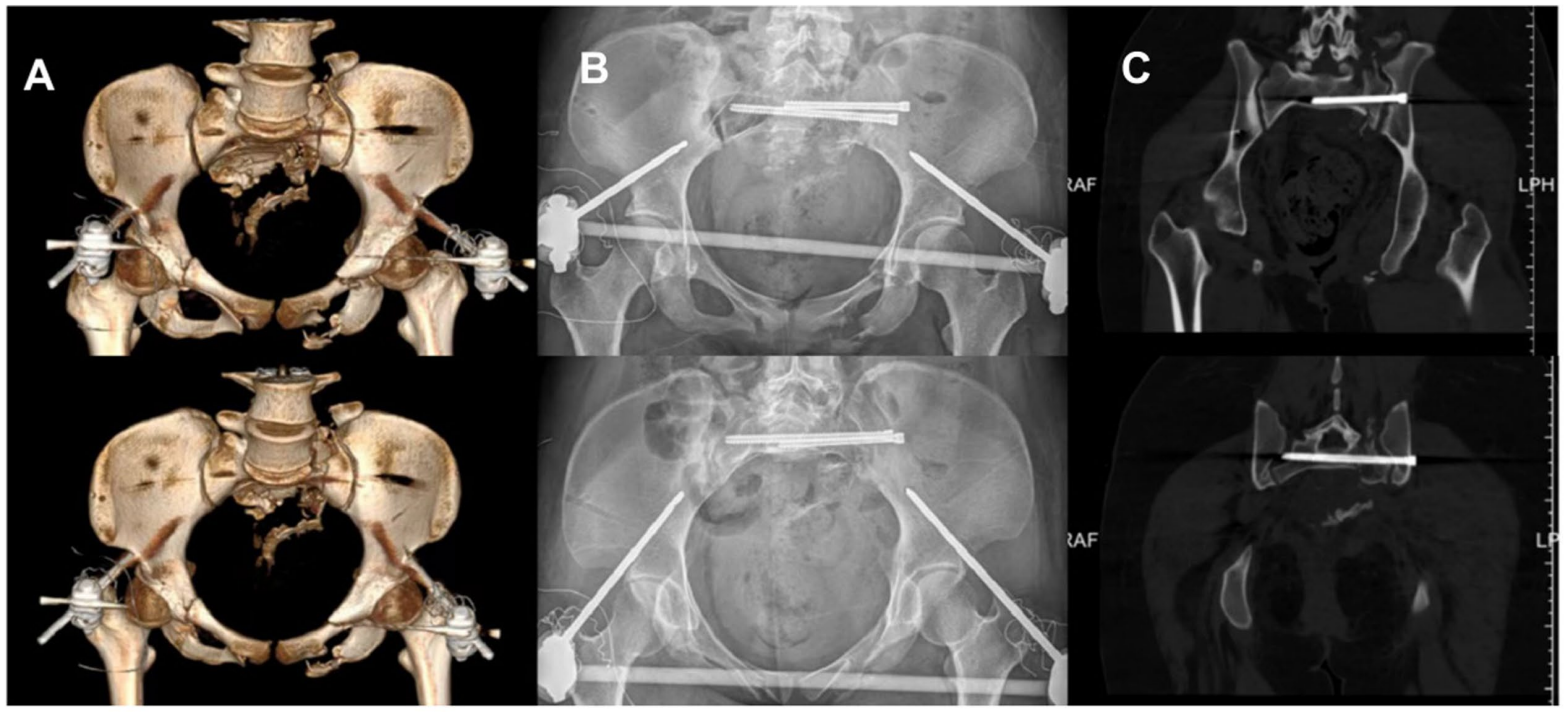
Postoperative X-ray and 3D CT images demonstrated the patient’s postoperative outcome. **(A)** Postoperative CT three dimensional reconstruction. **(B)** Postoperative pelvic X-ray orthopantomogram and entrance position. **(C)** Postoperative CT plain scan of the pelvis.

The fixation was performed using a standard S1 sacroiliac screw combined with an extended S2 sacroiliac screw. The anterior ring was stabilized with an anterior inferior iliac spine external fixator. Immediate postoperative X-ray showed restoration of the pelvic axis and accurate screw placement. One week postoperatively, CT confirmed fracture reduction meeting the excellent criteria of the Matta standard Figure 5. At the 3-month follow-up, the patient reported no pain, normal hip joint range of motion, partial recovery of lower limb sensation and muscle strength, and imaging demonstrated good fracture healing with no signs of internal fixation loosening or fracture. The patient reported, “I can sit normally now, and walk with double crutches without help. I can also do some light daily activities like making the bed. The numbness in my lower back and thighs has gotten much better, and I’m confident about getting back to my daily life soon.”

## 5 Discussion

Sacral vertical fractures are a type of posterior pelvic ring injury typically caused by high-energy trauma. These fractures present clinical management challenges, and surgical intervention is often required to restore pelvic ring stability. The key focus of treatment is the reduction and fixation of the posterior pelvic ring (22–24). Percutaneous sacroiliac screws have become one of the most commonly used fixation methods due to their minimally invasive nature, reduced blood loss, and lower risk of infection (25). This method is suitable for patients with relatively stable sacral fractures. However, for complex vertical fractures, a single sacroiliac screw may not provide sufficient stability, especially in cases with concomitant horizontal displacement.

Some studies have suggested that dual-plane sacroiliac screw fixation could be an effective strategy for enhancing sacral stability. This approach involves placing screws on two different planes to increase the fixation strength and stability of the fracture fragments. Research indicates that dual-plane fixation offers superior resistance to torsion and shear forces compared to single-plane fixation, particularly in cases of sacral fractures with vertical instability. Additionally, extending the length and varying the angles of the dual-plane screws can further optimize mechanical performance and improve surgical outcomes (8, 26).

Some studies have proposed different views. Schildhauer et al. (27) conducted a biomechanical study comparing early weight-bearing in patients with unstable sacral fractures. The study concluded that triangular fixation provides significantly greater stability for the posterior pelvic ring than sacroiliac screws. Triangular fixation combines lumbopelvic fixation with transverse stabilization of sacral fractures, allowing patients with type C pelvic fractures to immediately engage in full weight-bearing functional exercises (28).

For complex sacral vertical fractures, especially those with a high degree of instability, clinicians have begun exploring the combined use of long sacroiliac screws and iliolumbar fixation. Long sacroiliac screws extend across to the contralateral ilium, providing stabilization not only to the sacrum but also to the entire pelvis. Iliolumbar fixation offers additional support, particularly at the lumbopelvic junction, thereby enhancing the overall treatment outcomes. Studies have shown that this combined approach has distinct advantages in restoring sacral anatomy and maintaining pelvic stability, though it does increase the complexity of the surgery and the technical demands on the surgeon (29, 30).

In this patient case, dual-plane screw fixation and triangular fixation showed comparable efficacy. Dual-plane sacroiliac screw fixation was prioritized because it preserves the patient’s lumbar mobility to the greatest extent possible.

There are various methods for fixing sacral fractures, and selecting the optimal fixation technique is crucial for surgical success. This study integrated preoperative finite element analysis (FEA) to further optimize fixation strategies and surgical approaches, thereby achieving personalized treatment plans. In this patient case, the study focused on two biomechanically stable fixation methods suitable for unilateral sacral vertical fractures (Denis Type II, AO C1.3 type): dual-plane sacroiliac joint screw fixation and sacroiliac joint screw combined with iliac-lumbar fixation. The study designed and compared three fixation strategies, with results showing no statistically significant difference in biomechanical stability between dual-plane fixation and long sacroiliac screw combined with iliac-lumbar fixation. However, dual-plane fixation preserves lumbar mobility. Therefore, for this patient, dual-plane sacroiliac joint screw fixation should be prioritized to ensure a minimally invasive surgical approach.

With advancements in surgical technology, robotic-assisted techniques are increasingly being applied to the surgical management of sacral vertical fractures. Surgical robots improve the precision of screw placement, reduce operational errors, and minimize radiation exposure. However, for complex displaced sacral fractures, inadequate reduction significantly increases the risk of complications associated with sacroiliac screw fixation, making proper reduction a critical intraoperative factor. Recent studies have shown that using robotic systems for sacral fixation enhances surgical safety and accuracy—especially in the reduction and fixation of complex fractures. This study utilized a unique 3D image-guided intelligent RAFR system, which enables truly intelligent, minimally invasive reduction surgery for complex pelvic fractures.

The RAFR (robotic-assisted fracture reduction) system used in this study offers an innovative solution in this context. The RAFR system, based on three-dimensional imaging technology, provides enhanced precision and control for minimally invasive reduction of complex fractures through preoperative planning and intelligent navigation. Compared to traditional surgical methods, this system demonstrates significant advantages, including higher reduction accuracy and a lower rate of complications. In studies involving this RAFR system, 95.5% of 22 patients with unstable pelvic fractures achieved excellent or good reduction outcomes based on the Matta criteria, with the operation process causing no additional damage to patients, demonstrating favorable safety and effectiveness that meet clinical requirements (20).

The primary advantage of the RAFR system lies in its ability to use three-dimensional imaging guidance for reduction planning, minimizing judgment errors inherent in manual operations and enhancing the consistency of surgical quality. This technology allows for the personalized design of the optimal reduction path and target position, ensuring the most precise adjustments to the fracture site during surgery. Additionally, the automation capabilities of the RAFR system significantly reduce operative time, improve surgical efficiency, and provide clearer guidance for the surgeon, thereby lowering the risk of radiation exposure (31–35).

This study has several limitations: While this study explored the clinical application of FEA and robot-assisted pelvic reduction and used the patient’s own CT data to construct an FEA model (simulating the patient’s mechanical characteristics), the model can only partially explain features of the patient’s fracture and internal implants. It cannot fully restore the patient’s actual condition or completely represent the patient’s actual biomechanical status. Future research should continue to focus on improving the accuracy of FEA models. Additionally, the RAFR system also has limitations in practical application. The system’s path planning relies on pelvic symmetry, which may not provide sufficient reduction accuracy for patients with bilateral severe comminuted pelvic fractures. This reliance on the symmetry assumption poses challenges when dealing with complex asymmetric fractures. Furthermore, the RAFR system currently lacks the ability to adjust the reduction path in real-time during surgery, which may limit its effectiveness in certain complex cases. Although the RAFR system offers significant clinical advantages in minimally invasive treatment of pelvic fractures, further research is needed to optimize its technical performance and expand its scope of application. Future research directions may include enhancing the system’s automatic path adjustment capabilities, developing technical solutions applicable to a broader range of fracture types, and conducting large-scale randomized controlled trials to further validate its clinical efficacy and safety.

## 6 Conclusion

This case study demonstratively illustrates the application value of combining finite element analysis with a robot-assisted fracture reduction system in personalized treatment through the management of a complex sacral fracture complicated by multiple injuries. The innovation of this approach lies in the seamless integration of preoperative biomechanical simulation and intraoperative robotic precision: (1) FEA evaluates the biomechanical properties of different fixation schemes, providing a scientific basis for developing individualized surgical plans; (2) the robotic-assisted system uses 3D imaging navigation and precise manipulation to achieve minimally invasive, precise reduction of complex fractures. This effectively overcomes the challenge of insufficient surgical precision in anatomically complex regions—a limitation of traditional surgery. Clinical practice has demonstrated that this technological combination not only enhances the rationality of treatment decisions through preoperative optimization but also reduces trauma and ensures treatment efficacy through intraoperative precise manipulation, without any related complications. It provides a feasible technical pathway for personalized, minimally invasive treatment of complex sacral fractures, and its application potential warrants further exploration and promotion.

## Data Availability

The original contributions presented in the study are included in the article/Supplementary material, further inquiries can be directed to the corresponding author.
