# Supplementary material for: Case Report: Robot-assisted sacral fracture reduction with patient-specific finite element planning
Source: Front Med (Lausanne). 2025 Nov 14;12:1710981. doi: 10.3389/fmed.2025.1710981 (PMC12660070; doi:10.3389/fmed.2025.1710981)
Supplement: Supplementary file 1 [file Supplementary_file_1.docx]

1. **Finite element modelling on modelling and material settings**

This study utilized CT data of the sacral bone, L4-L5 vertebral bodies, and pelvis from the patient (64-slice spiral CT, 1 mm slice thickness). Using image processing software (Mimics 21.0), virtual three-dimensional models of the lumbar spine and pelvis were created from the DICOM-format CT data. Components were segmented based on CT grey values. The pelvic 3D model generated in Mimics was imported into Design X 2020.0 software for smoothing to make the model suitable for computation. Ten-node tetrahedral elements were used. The mesh model was then assigned material properties, set as heterogeneous and isotropic. Material properties were assigned to different skeletal regions based on grey values, with the pelvic grey values divided into ten levels using the built-in formula in Mimics.

The material parameters for specific bone components were determined using Mimics' built-in equations and CT gray value classification techniques. The CT value range for cancellous bone was set between 101 and 816, while cortical bone was defined for CT values above 816. This approach eliminated the need to distinguish the boundary between cortical and cancellous bone, thereby reducing modeling time. The relationship between bone density, elastic modulus, and CT values was then established using the following empirical equations:

$$\rho=0.047+1.122\times10^{-3} \text{HU} \text{HU}\leq816$$

$$\rho=7.69\times10^{-4} \text{HU}+1.028 \text{HU}\geq816$$

$$\begin{matrix} E=1904\rho^{1.64} \text{(Trabecular Bone)} \\ E=2065\rho^{3.09} \text{(Cortical Bone)} \end{matrix}$$

In the equation, ρ and E represent the density and elastic modulus of a specific region of the bone tissue in the CT image, respectively, with units of g/cm³ and MPa. CT denotes the CT value at that specific point.The Poisson’s ratio was set to 0.3 for cortical bone and 0.2 for Trabecular bone.[1-3]

Use the design functions in SpaceClaim 2019 software to draw models such as intervertebral discs and cylindrical screws, then use the move command to move the drawn models to the appropriate position to simulate fracture reduction and fixation.

Spring elements were used to simulate the main ligaments (sacroiliac ligament, anterior sacroiliac ligament, posterior long sacroiliac ligament, posterior short sacroiliac ligament, interosseous sacroiliac ligament, sacral tubercle ligament, pubic arch ligament, etc.), set the origin and destination points and stiffness values according to anatomical location, Table 1. [4-7]

Table 1 Material properties in the finite element model

| Project | Stiffness value (N/mm) | Number of units (roots) |
| --- | --- | --- |
| Sacroiliac anterior ligament | 700 | 10X2 |
| Sacroiliac posterior ligament long | 1000 | 4X2 |
| Sacroiliac posterior ligament short | 400 | 10X2 |
| Sacroiliac interosseous ligament | 2800 | 4X2 |
| Sacral spinous ligament | 1400 | 5X2 |
| Sacral tubercle ligament | 1500 | 5X2 |
| Pubic superior ligament | 500 | 1X1 |
| Pubic arch ligament | 500 | 1X1 |
| Iliac lumbar ligament | 1000 | 4X2 |

The elastic modulus and Poisson's ratio of materials such as cortical bone, cancellous bone, articular cartilage, intervertebral discs, pubic discs, and titanium alloy internal fixators are referenced from Table 2[4-7]. Sacral cartilage thickness is 1.8 mm, iliac cartilage thickness is 0.9 mm, and the spacing is 0.3 mm[8] .

Table 2 Material properties in the finite element model

| Material | modulus of elasticity（MPa） | Poisson's ratio |
| --- | --- | --- |
| Pubic disc | 5 | 0.45 |
| Articular cartilage | 10 | 0.4 |
| Posterior spinal structure | 3500 | 0.25 |
| Titanium screws | 110000 | 0.3 |
| Fibrous ring fibres | 450 | 0.3 |
| Vertebral endplates | 1000 | 0.4 |
| Disc nucleus pulposus | 1 | 0.45 |
| Fibrous ring matrix | 4.2 | 0.3 |
| Cartilage endplates | 19 | 0.3 |

All bone structures and internal fixations were meshed using 10-node tetrahedral cells, with a total of 233768 cells and 1092128 nodes in the complete pelvis model.

The sacroiliac joint cartilage surface is set as a sliding friction contact (friction coefficient 0.015)[9] . The fracture surface is set as a sliding friction contact (friction coefficient 0.3) [10]. The screw thread and bone contact surface, the connection between the screw tail and the disc, and the disc and cortical bone surface are all bound connections.

1. **Data and statistical analysis of the displacement of the upper surface of the sacrum**

Specifically the displacement of each observation point on the upper surface of the sacrum for each model is shown in Table 3.We performed normality test on the data The data of each group satisfy the normal distribution, Table 4.The data of each group were tested by variance chi-square test, Table 5.ANOVA test was performed on the data of each group, and the results show that there is no statistical significance in the difference between the data of each group，Table6.

Table 3 Displacement data for the superior surface of the sacrum

| Groups | A（mm） | B（mm） | C（mm） | D（mm） | E（mm） |
| --- | --- | --- | --- | --- | --- |
| SDS1EDS2 | 0.64003 | 0.56687 | 0.68932 | 0.73965 | 0.65342 |
| L5SDS1 | 0.58271 | 0.56515 | 0.67418 | 0.69285 | 0.61795 |
| L5EDS2 | 0.61334 | 0.61633 | 0.72263 | 0.73052 | 0.65944 |

Table 4 Normality test for four groups of data

| **Tests of Normality** | | | | | | | |
| --- | --- | --- | --- | --- | --- | --- | --- |
|  | FEA | Kolmogorov-Smirnov^a^ | | | Shapiro-Wilk | | |
|  |  | Statistic | df | Sig. | Statistic | df | Sig. |
| displacement | 1 | .190 | 5 | .200^*^ | .986 | 5 | .962 |
|  | 2 | .203 | 5 | .200^*^ | .920 | 5 | .531 |
|  | 3 | .233 | 5 | .200^*^ | .849 | 5 | .192 |
| *. This is a lower bound of the true significance. | | | | | | | |
| a. Lilliefors Significance Correction | | | | | | | |

Table 5 **Test of Homogeneity of Variances**

| **Test of Homogeneity of Variances** | | | | | |
| --- | --- | --- | --- | --- | --- |
|  | | Levene Statistic | df1 | df2 | Sig. |
| displacement | Based on Mean | .002 | 2 | 12 | .998 |
|  | Based on Median | .001 | 2 | 12 | .999 |
|  | Based on Median and with adjusted df | .001 | 2 | 10.496 | .999 |
|  | Based on trimmed mean | .002 | 2 | 12 | .998 |

Table 6 ANOVA test

| **ANOVA** | | | | | |
| --- | --- | --- | --- | --- | --- |
| displacement | | | | | |
|  | Sum of Squares | df | Mean Square | F | Sig. |
| Between Groups | .005 | 2 | .002 | .688 | .521 |
| Within Groups | .041 | 12 | .003 |  |  |
| Total | .046 | 14 |  |  |  |

References

[1] Rho JY, Hobatho MC, Ashman RB. Relations of mechanical properties to density and CT numbers in human bone. Med Eng Phys. 1995;17(5):347–55.

[2] Peng L, Bai J, Zeng X, Zhou Y. Comparison of isotropic and orthotropic material property assignments on femoral finite element models under two loading conditions. Med Eng Phys. 2006;28(3):227–33.

[3] Zhang L, Peng Y, Du C, Tang P. Biomechanical study of four kinds of percutaneous screw fixation in two types of unilateral sacroiliac joint dislocation: a finite element analysis. Injury. 2014;45(12):2055–9.

[4] Lu YM, Hutton WC, Gharpuray VM. The effect of fluid loss on the viscoelastic behavior of the lumbar intervertebral disc in compression. J Biomech Eng. 1998;120(1):48–54.

[5] Faizan A, Sairyo K, Goel VK, Biyani A, Ebraheim N. Biomechanical rationale of ossification of the secondary ossification center on apophyseal bony ring fracture: a biomechanical study. Clin Biomech (Bristol). 2007;22(10):1063–7.

[6] Zhao Y, Li J, Wang D, Liu Y, Tan J, Zhang S. Comparison of stability of two kinds of sacro-iliac screws in the fixation of bilateral sacral fractures in a finite element model. Injury. 2012;43(4):490–4.

[7] Zhao Y, Zhang S, Sun T, Wang D, Lian W, Tan J, et al. Mechanical comparison between lengthened and short sacroiliac screws in sacral fracture fixation: a finite element analysis. Orthop Traumatol Surg Res. 2013;99(5):601–6.

[8] Lu Y, He Y, Li W, Yang Z, Peng R, Yu L. Comparison of Biomechanical Performance of Five Different Treatment Approaches for Fixing Posterior Pelvic Ring Injury. J Healthc Eng. 2020;2020:5379593.

[9] Hu P, Wu T, Wang HZ, Qi XZ, Yao J, Cheng XD, et al. Biomechanical Comparison of Three Internal Fixation Techniques for Stabilizing Posterior Pelvic Ring Disruption: A 3D Finite Element Analysis. Orthop Surg. 2019;11(2):195–203.

[10] Hartensuer R, Grüneweller N, Lodde MF, Evers J, Riesenbeck O, Raschke M. The S2-Alar-Iliac Screw for Pelvic Trauma. Z Orthop Unfall. 2021;159(5):522–32.
